# Gender difference following high cholesterol diet induced renal injury and the protective role of rutin and ascorbic acid combination in Wistar albino rats

**DOI:** 10.1186/1476-511X-11-41

**Published:** 2012-03-16

**Authors:** Salim Salih Al-Rejaie, Hatem Mustafa Abuohashish, Osama Abdelrahman Alkhamees, Abdulaziz Mohammed Aleisa, Abdulaziz S Alroujayee

**Affiliations:** 1 Department of Pharmacology and Toxicology, College of Pharmacy, P.O. Box 2457, King Saud University, Riyadh 11451, Saudi Arabia; 2 Department of Pharmacology, College of Medicine, Al-Imam University, Riyadh, PO Box 11623, Saudi Arabia; 3 College of Medicine, Al-Imam University, Riyadh, PO Box 11623, Saudi Arabia

**Keywords:** High cholesterol diet, Nephrotoxicity, Renal injury, Gender difference, Rutin, Ascorbic acid

## Abstract

**Background:**

An increased interest is given to the impact of high fat diet on health worldwide. Abnormalities in lipid metabolism induced by high cholesterol diet (HCD) were reported to exacerbate renal diseases via oxidative stress pathways. Rutin and ascorbic acid showed a protective role against oxidative stress-mediated diseases. Furthermore, both lipid metabolism and tissue response to oxidative stress damage was found to vary according to animal gender. Thus, the objective of this work was to examine possible gender-related differences and the possible protective effects of rutin and ascorbic acid supplementation on high cholesterol diet induced nephrotoxicity.

**Methods:**

96 young male and female Wistar albino rats were used. HCD supplemented animals were treated with rutin alone or in combination with ascorbic acid for 6 weeks. Creatinine plasma level was estimated. Furthermore, kidney levels of nucleic acids, total protein, malondialdehyde (MDA), reduced glutathione (GSH), total cholesterol, and triglycerides were determined. Finally, kidney tissues were used for histopathological examination.

**Results:**

HCD supplementation decreased kidney level of nucleic acids, which was more prominent in female animals. Both vitamin combination significantly attenuated HCD induced decrease in nucleic acids. Moreover, kidney level of MDA was significantly altered by HCD in both genders, which was inhibited by rutin and ascorbic acid alone or in combination in male groups and by both vitamins in female groups. There was a reduction in kidney level of GSH by HCD, especially in male groups, which was attenuated by rutin and ascorbic acid combination. Kidney levels of total cholesterol and triglycerides were significantly increased by HCD supplementation in both genders. Coadministration with rutin and/or ascorbic acid protected from such increase, which was more obvious in both vitamins combination. Histopathological investigation supported vitamins protective effect, which was more prominent in male vitamins combination group.

**Conclusions:**

HCD-induced renal injury in female was higher than in male animals, suggesting a better anti-oxidative stress defense response in male's kidney. Moreover, the antioxidant and reno-protective effects of rutin and ascorbic acid were augmented following their combination.

## Introduction

It is well known that lifestyle and diet play a role in the development of kidney disease. Several studies indicated that abnormalities in lipid metabolism can often accompany and exacerbate renal disease [1,2]. Hypercholesterolemia is well-known to be an independent risk factor for renal injury [3] and to aggravate the pathogenesis of a variety of clinical and experimental renal diseases [4-6]. High cholesterol diet (HCD) was found to increase blood pressure and to induce renal injury [7]. Moreover, many accumulating evidences support the idea that HCD exacerbates kidney damage in animal models of kidney disease [8]. Previous data showed that even a short exposure to HCD supplementation is associated with an increase in oxidative stress and renal inflammation [9]. Indeed, HCD supplementation to animals was reported to significantly increase kidney oxidative stress parameter and to significantly reduce kidney antioxidant parameters [10]. Therefore, the inhibition of oxidative stress under hypercholesterolemic conditions is considered to be an important therapeutic approach for kidney related diseases.

Rutin (RT), a quercetin-3-rutinosid or vitamin-P, is considered as one of flavonoid glycosides, which is found in onions, apples, tea and red wine [11]. Rutin is well known to exhibit multiple pharmacological activities including antibacterial, antitumor, anti-inflammatory, anti-diarrheal, antiulcer, anti-mutagenic, vasodilator and immunomodulator [12]. Furthermore, rutin showed an inhibitory effect against membrane lipid peroxidation [13]. Rutin was found to have renal-protective effects via its antioxidant activities which suggest its protective role in oxidative stress-mediated diseases [13-15]. Vitamin C is a water-soluble enzyme cofactor, abundantly present in different plants and some animals. It is present in two chemical forms: the reduced form (ascorbic acid; AA) and the oxidized form (dehydroascorbic acid; DHA). AA is the most predominant form in the human body and is involved in tissue growth and repair. AA has a potent antioxidant activity and is well known to protect tissues from oxidative injury through efficiently quenching the damaging free radicals produced by many biological processes [16,17].

Gender difference currently plays an important role in the etiology of hyperlipidaemic-induced disorder including cardiovascular diseases (CVD). For instance, men are more susceptible to coronary heart disease than age-matched women. However, postmenopausal women have an equal chance of CVD with men [18,19]. The underlying mechanisms for this difference are related to the known effects of estrogens of lipid metabolism, such as a decrease in HDL catabolism via a decrease in hepatic lipase activity and an increase in LDL catabolism via an increase in the number of LDL receptors [20]. Moreover, the severity of oxidative stress tissue damage and injury may vary according to gender difference. Hepatic oxidative stress and inflammatory response in acute uremia after bilateral nephrectomy was shown to be more significant in female than male rats [21]. Sex dimorphism in pancreas oxidative stress as response to HCD was also reported, where female rats were protected against oxidative damage [22]. Furthermore, application of chronic mild stress to male and female rats was shown to induce different oxidative stress and compensatory responses, which was suggested to be due to differences in the mechanisms regulating oxidant/antioxidant pathways [23].

The current study was designed with major two goals; (1) to investigate gender-related differences in response to high cholesterol diet induced nephrotoxicity using Wistar albino rats as an animal model; (2) to evaluate the potential beneficial effects of rutin and/or ascorbic acid supplementation on high cholesterol diet induced nephrotoxicity.

## Materials and methods

### Materials

#### Animals

Young male and female Wistar albino rats (total animals = 96 rats) were provided by the Experimental Animal Care Center (King Saud University, Riyadh, Saudi Arabia). Animals were roughly the same age, weighing 80-100 grams. Animal environment was maintained under controlled conditions of temperature (22 ± 1°C), humidity (50-55%), and light (12 h light/dark cycles). All methods including euthanasia procedure were conducted in accordance with the Guide for the Care and Use of Laboratory Animals, Institute for Laboratory Animal Research, National Institute of Health (NIH Publications No. 80-23; 1996) and approved by the Ethical Guidelines of the Experimental Animal Care Center (College of Pharmacy, King Saud University, Riyadh, Saudi Arabia).

#### Diet

All animals' diets were prepared in pellet form by adding test materials in rat chow powder following shade dry method. Table 1 show diet contents for each group. All the diets were prepared weekly and shade dried. During whole experimental period, all groups of animals were kept on free access to food and water.

**Table 1:** Diet contents for each group

Group	Diet contents
**Control**	rat chow

**RT**	rat chow + 0.2% rutin

**AA**	rat chow + 0.4% ascorbic acid

**RT + AA**	rat chow + 0.1% RT + 0.2% AA

**HCD**	rat chow + 1% cholesterol and 0.5% cholic acid

**RT + HCD**	rat chow + 0.2% RT + 1% cholesterol and 0.5% cholic acid

**AA + HCD**	rat chow + 0.4% AA + 1% cholesterol and 0.5% cholic acid

**RT + AA + HCD**	rat chow + 0.1% RT + 0.2% AA + 1% cholesterol and 0.5% cholic acid

### Methods

#### Experimental design and animal grouping

All animals were randomly divided in to eight groups (six male and six female rats per each group) as follow; group-1: Control, group-2: RT, group-3: AA, group-4: RT + AA, group-5: HCD, group-6: RT + HCD, group-7: AA + HCD, group-8: RT + AA + HCD. The experimental diets were supplemented for 6 consecutive weeks. At end of the 6th week rats were sacrificed by decapitation and the trunk blood was collected in heparinized tubes. Both kidneys were dissected and weighed. Kidney tissues were immediately dipped in liquid nitrogen for 1 min and then preserved at -75°C (Ultra-low freezer, Environmental Equipment, Cincinnati, Ohio, USA) till analysis. The plasma was collected after centrifugation at 4,000 rpm for 15 min and stored in freezer at -20°C till analysis.

#### Determination of plasma levels of creatinine

Creatinine concentrations were estimated in plasma samples by using commercially available diagnostic kits (Human, Wiesbaden, Germany).

#### Determination of kidney levels of nucleic acids and total protein

Kidney levels of nucleic acids (DNA and RNA) were estimated by the method described by Bregman, 1983 [24]. Kidney tissues were homogenized in ice-cold distilled water and the homogenates were suspended in 10% ice-cold trichloroacetic acid (TCA). Pellets were extracted with 95% ethanol twice. DNA levels were determined by treating the nucleic acid extract with diphenylamine reagent and measuring the intensity of blue color at 600 nm. For quantification of RNA, the nucleic acid extract was treated with orcinol reagent and the green color was recorded at 660 nm on spectrophotometer (LKB-Pharmacia, Mark II, Ireland). The modified Lowry method by Schacterle and Pollack [25] was used to estimate kidney levels of total protein. Bovine plasma albumin was used as standard.

#### Determination of kidney levels of MDA

Kidney levels of MDA were estimated as described by Ohkawa et al., 1979 [26]. Briefly, kidney tissue was homogenized in aqueous 0.15 M KCl solution to give 10% homogenate. One ml of homogenate was then mixed with one ml of 10% trichloroacetic acid (TCA) and centrifuged at 3,000 rpm for 15 min. One ml of supernatant was suspended into one ml of 0.67% 2-thiobarbutaric acid. Sample tubes were then placed into a boiling water bath and kept for 15 min. Samples were allowed to cool down at room temperature followed by centrifugation at 3,000 rpm for 15 min. The Optical density of the clear pink supernatants was measured at 532 nm.

#### Determination of kidney levels of GSH

The method prescribed by Sediak and Lindsay, 1968 [27] was used to determine the concentration of glutathione (GSH) in the kidney. Briefly, a cross section of kidney tissue were dissected out and homogenized in ice-cold 0.02 M ethylenediaminetetraacetic acid (EDTA). An aliquots of 0.5 mL of tissue homogenate was mixed with 0.2 M Tris buffer, pH 8.2 and 0.1 mL of 0.01 M Ellman's reagent, [5,5'-dithiobis-(2-nitro-benzoic acid)] (DTNB). Each sample tube was centrifuged at 3,000 g at room temperature for 15 min. The absorbance of the clear supernatants was measured using spectrophotometer at 412 nm in one centimeter quarts cells.

#### Determination of kidney levels of total cholesterol and triglycerides

Lipids contents of kidney tissues were extracted using the method described by Folch et al., 1957 [28]. In brief, kidney tissues were homogenized in 0.15 mol/L of ice-cold KCl (10% w/w) and lipids were extracted with chloroform: methanol (2:1). After the extraction and evaporation, tissue lipids were re-dissolved in isopropanol, and kidney cholesterol and triglyceride levels were estimated enzymatically by commercially available kits (Human, Wiesbaden, Germany).

#### Histopathological evaluation of the kidneys

One half of kidney from control, HCD, RT + HCD, AA + HCD and RT + AA + HCD male and female groups was removed for histopathological examination. Kidney tissues were fixed in 10% neutral buffered formalin, embedded in paraffin wax, sectioned at 3 μm, stained with Hematoxylin and Eosin (H & E) stain and placed in slides for light microscopic examination. To avoid any type of bias, the slides were coded and examined by a histopathologist who was blinded to the treatment groups. Grading of kidney injury was preformed according to Table 2. The degree of the nephrotoxicity was considered according to the total score of kidney injury grading where; (0-1) classified as no nephrotoxicity, (2-4) classified as mild nephrotoxicity, (5-7) classified as moderate nephrotoxicity and (8-10) classified as sever nephrotoxicity.

**Table 2:** Histopathological grading of kidney injury

Type of damage	Score
GLOMERULAR DAMAGE:	
o None	0
o Less than 25% of glomeruli are involved	1
o 25-50% of glomeruli are involved	2
o 51-75% of glomeruli are involved	3
o More than 75% of glomeruli are involved	4

ACUTE TUBULAR NECROSIS	
o None	0
o Less than 25% of renal tubules are involved	1
o 25-50% of renal tubules are involved	2
o 51-75% of renal tubules are involved	3
o More than 75% of renal tubules are involved	4

TUBULOINTERSTITIAL NEPHRITIS	
o None	0
o Leukocytes confined within the interstitium	1
o Leukocytes within the interstitium and tubular epithelial cells	2

#### Statistical analysis

All data were expressed as mean ± Standard Deviation (SD). Data were statistically analyzed using one-way ANOVA followed by Student-Newman-Keuls multiple comparisons test. The differences were considered statistically significant at *P *< 0.05. GraphPad prism program (version 5) was used as analyzing software.

## Results

### Effects on kidney weight

Kidney weights did not show significant difference between control group and other treated groups in both male and female animals (Table 3). HCD vitamins treated male and female groups (RT + HCD, AA + HCD and RT + AA + HCD) did not have kidney weights that differ significantly from their respective control vitamins supplemented male and female groups (RT, AA and RT + AA, respectively). Similarly, HCD vitamins treated male and female groups (RT + HCD, AA + HCD and RT + AA + HCD) did not show kidney weights that differ significantly from male and female HCD administered groups (Table 3).

**Table 3:** Effect of rutin (RT) and/or ascorbic acid (AA) on kidney weight in high-cholesterol diet (HCD) fed rats following 6 weeks of supplementation

Treatment (in rat chow)	Kidney (g)/100 g body weight	
	
	Male	Female
**Control**	0.75 ± 0.07	0.69 ± 0.05

**RT (0.2%)**	0.71 ± 0.06	0.70 ± 0.04

**AA (0.4%)**	0.76 ± 0.04	0.74 ± 0.08

**RT (0.1%) + AA (0.2%)**	0.78 ± 0.04	0.70 ± 0.04

**HCD (1% cholesterol + 0.5% cholic acid)**	0.72 ± 0.04	0.73 ± 0.16

**RT (0.2%) + HCD**	0.72 ± 0.03	0.68 ± 0.04

**AA (0.4%) + HCD**	0.74 ± 0.07	0.72 ± 0.10

**RT (0.1%) + AA (0.2%) + HCD**	0.75 ± 0.07	0.74 ± 0.08

Data were expressed as Mean ± S.D and analyzed using one-way ANOVA followed by Student Newman-Keuls method as post hoc test. Six rats were used in each group

#### Effects on plasma creatinine

Creatinine plasma level in both male and female rats was not significantly altered at any group as compared to control groups (Table 4). Furthermore, there was no significant difference between HCD vitamins treated male and female animals (RT + HCD, AA + HCD and RT + AA + HCD) and their respective control vitamins supplemented groups (RT, AA and RT + AA, respectively). Creatinine plasma level was not significantly different in HCD vitamins treated groups (RT + HCD, AA + HCD and RT + AA + HCD) as compared to HCD administered rats in both male and female groups (Table 4).

**Table 4:** Effect of rutin (RT) and/or ascorbic acid (AA) on plasma level of creatinine in high-cholesterol diet (HCD) fed rats following 6 weeks of supplementation

Treatment (in rat chow)	Creatinine (μmol/l)	
	
	Male	Female
**Control**	51.94 ± 12.59	35.59 ± 9.71

**RT (0.2%)**	49.76 ± 6.94	32.91 ± 14.13

**AA (0.4%)**	51.21 ± 4.52	35.21 ± 12.03

**RT (0.1%) + AA (0.2%)**	55.21 ± 2.98	44.77 ± 14.21

**HCD (1% cholesterol + 0.5% cholic acid)**	43.95 ± 6.53	36.35 ± 13.88

**RT (0.2%) + HCD**	54.12 ± 9.29	47.07 ± 16.54

**AA (0.4%) + HCD**	55.93 ± 20.93	42.10 ± 11.40

**RT (0.1%) + AA (0.2%) + HCD**	53.39 ± 12.84	37.12 ± 9.79

Data were expressed as Mean ± S.D and analyzed using one-way ANOVA followed by Student Newman-Keuls method as post hoc test. Six rats were used in each group

#### Effects on nucleic acids and total protein

In male groups, kidney levels of both nucleic acids and total protein were not significantly altered by HCD administration either alone or with vitamins as compared to control group (Table 5). DNA kidney level was significantly decreased in HCD vitamins treated male groups (RT + HCD, AA + HCD and RT + AA + HCD) as compared to their respective control groups (RT, AA and RT + AA, respectively), while kidney level of RNA and total protein were not significantly altered. Moreover, no significant differences were found between HCD vitamins treated male animals (RT + HCD, AA + HCD and RT + AA + HCD) and HCD-fed male group (Table 5). In female groups, kidney levels of DNA were significantly reduced in HCD, RT + HCD and AA + HCD groups as compared to control group. Kidney RNA levels in female group were also significantly reduced by HCD administration either alone or in combination with RT and/or AA as compared to control animals. Such reduction was not demonstrated in total protein of female groups. Indeed, a significant difference in both nucleic acids and total protein was found between RT + HCD as well as AA + HCD treated female rats and their respective vitamin supplemented control groups (RT and AA). Combination of rutin and ascorbic acid with HCD (RT + AA + HCD) in female group did not show significant difference from RT + AA group in kidney's DNA and total protein, while RNA level was significantly different in these two groups. There was no significant difference between HCD female group and HCD supplemented with rutin and/or ascorbic acid (RT + HCD, AA + HCD and RT + AA + HCD) in kidney levels of DNA and total protein. Similar effect was observed in RNA kidney level except for RT + AA + HCD group, which was significantly different from HCD female group (Table 5).

**Table 5:** Effect of rutin (RT) and/or ascorbic acid (AA) on kidney nucleic acids and total protein levels in high-cholesterol diet (HCD) fed rats following 6 weeks of supplementation

Treatment (in rat chow)	DNA (μg/100 mg)		RNA (μg/100 mg)		Total protein (mg/100 mg)	
	
	Male	Female	Male	Female	Male	Female
**Control**	101.35 ± 18.33	110.56 ± 17.93	266.30 ± 26.60	240.16 ± 19.00	12.21 ± 0.47	11.26 ± 0.63

**RT (0.2%)**	108.33 ± 24.25	117.22 ± 12.08	271.22 ± 27.50	254.10 ± 11.80	12.15 ± 0.49	11.63 ± 0.43

**AA (0.4%)**	110.25 ± 18.05	114.21 ± 21.35	278.77 ± 17.28	248.52 ± 13.58	11.84 ± 0.53	11.95 ± 0.24

**RT (0.1%) + AA (0.2%)**	114.35 ± 17.37	112.60 ± 21.77	269.24 ± 31.53	250.20 ± 13.05	12.20 ± 0.60	11.53 ± 0.56

**HCD (1% cholesterol + 0.5% cholic acid)**	76.07 ± 15.99	75.64 ± 16.37 ^a^	247.01 ± 13.17	186.95 ± 19.04 ^a^	11.45 ± 0.35	10.64 ± 0.35

**RT (0.2%) + HCD**	81.45 ± 10.83 ^b^	79.63 ± 12.47 ^ab^	252.52 ± 20.72	192.11 ± 13.22 ^ab^	11.54 ± 0.28	10.80 ± 0.31 ^b^

**AA (0.4%) + HCD**	79.62 ± 11.54 ^c^	84.46 ± 9.00 ^ac^	255.66 ± 18.87	193.82 ± 20.91 ^ac^	11.76 ± 0.37	10.96 ± 0.15 ^c^

**RT (0.1%) + AA (0.2%) + HCD**	86.64 ± 11.89 ^d^	102.49 ± 14.25 ^e^	258.09 ± 21.65	219.12 ± 15.86 ^ade^	11.87 ± 0.31	11.15 ± 0.24

Data were expressed as Mean ± S.D and analyzed using one-way ANOVA followed by Student Newman-Keuls method as post hoc test. Six rats were used in each group. ^a^All treated groups vs. Control; ^b^RT + HCD vs RT group; ^c^AA + HCD vs AA group; ^d^RT + AA + HCD vs RT + AA group; ^e^RT + HCD, AA + HCD and RT + AA + HCD vs HCD

#### Effects on MDA and GSH

HCD administration to Wistar albino rats for 6 weeks significantly elevated kidney level of MDA in HCD male and female groups as well as in HCD vitamins treated female groups (RT + HCD, AA + HCD and RT + AA + HCD) as compared to control groups (Table 6). Kidney levels of MDA were also significantly high in HCD vitamins treated female animals (RT + HCD, AA + HCD and RT + AA + HCD) as compared to their respective control vitamins supplemented groups (RT, AA and RT + AA), which were not observed in male groups. Furthermore, HCD groups supplemented with rutin and/or ascorbic acid (RT + HCD, AA + HCD and RT + AA + HCD) did not have a significant difference in kidney level of MDA as compared to HCD group in male animals. In female animals, HCD group supplemented with rutin and ascorbic acid (RT + AA + HCD) expressed significant difference in kidney level of MDA as compared to HCD group, while (RT + HCD and AA + HCD) groups did not show such significant difference (Table 6). On the other hand, kidney levels of GSH were significantly decreased in HCD, RT + HCD and AA + HCD treated male groups, but not RT + AA + HCD group, as compared to control groups. Such significant decrease was not seen in female animals. Moreover, RT + HCD and AA + HCD treated male animals had a significantly reduced level of kidney GSH compared to their control vitamins supplemented groups (RT and AA, respectively). Female group supplemented with rutin and HCD was the only HCD vitamin treated group that had a significantly reduced GSH level in kidney as compared to its respective control vitamin treated group, RT group. Additionally, concurrent administration of AA or RT + AA with HCD significantly increased GSH kidney level in male groups as compared to HCD male group. Such effect was not seen in female groups (Table 6).

**Table 6:** Effect of rutin (RT) and/or ascorbic acid (AA) on kidney MDA and GSH levels in high-cholesterol diet (HCD) fed rats following 6 weeks of supplementation

Treatment (in rat chow)	MDA (mmol/g)		GSH (nmol/100 mg)	
	
	Male	Female	Male	Female
**Control**	168.77 ± 22.10	178.80 ± 15.99	131.68 ± 14.04	110.84 ± 18.16

**RT (0.2%)**	175.40 ± 19.88	184.13 ± 25.61	129.19 ± 9.45	122.87 ± 24.24

**AA (0.4%)**	164.19 ± 18.62	186.53 ± 25.54	128.14 ± 15.03	125.35 ± 39.96

**RT (0.1%) + AA (0.2%)**	161.67 ± 12.66	185.44 ± 29.42	126.87 ± 12.26	134.76 ± 15.95

**HCD (1% cholesterol + 0.5% cholic acid)**	204.91 ± 26.35 ^a^	253.80 ± 15.06 ^a^	94.93 ± 11.91 ^a^	79.56 ± 11.65

**RT (0.2%) + HCD**	194.50 ± 18.43	235.69 ± 11.26 ^ab^	98.29 ± 9.85 ^ab^	82.71 ± 13.96 ^b^

**AA (0.4%) + HCD**	182.48 ± 14.50	230.15 ± 13.94 ^ac^	101.28 ± 12.98 ^ace^	91.79 ± 12.32

**RT (0.1%) + AA (0.2%) + HCD**	164.44 ± 16.49	220.06 ± 16.02 ^ade^	117.71 ± 14.37 ^e^	106.01 ± 19.25

Data were expressed as Mean ± S.D and analyzed using one-way ANOVA followed by Student Newman-Keuls method as post hoc test. Six rats were used in each group. ^a^All treated groups vs. Control; ^b^RT + HCD vs RT group; ^c^AA + HCD vs AA group; ^d^RT + AA + HCD vs RT + AA group; ^e^RT + HCD, AA + HCD and RT + AA + HCD vs HCD

#### Effects on total cholesterol and triglycerides

In both male and female groups, HCD administration resulted in a significant increase in kidney levels of total cholesterol in all HCD-supplemented groups (Table 7). Moreover, kidney levels of total cholesterol in HCD vitamins treated male and female animals (RT + HCD, AA + HCD and RT + AA + HCD) were significantly different from their levels in vitamins supplemented control groups (RT, AA and RT + AA). In both genders, kidney level of total cholesterol was significantly low in HCD vitamins treated groups (RT + HCD, AA + HCD and RT + AA + HCD) as compared to HCD administered animals (Table 7). Kidney levels of triglycerides in male and female groups were significantly increased in HCD, RT + HCD and AA + HCD groups as compared to control groups. However, triglycerides were no significantly different in RT + AA + HCD and control groups in both male and female animals. In both male and female animals, there was a significant difference in kidney levels of triglycerides in RT + HCD and AA + HCD groups and their vitamins treated control animals in RT and AA groups, respectively. However, male and female rats' kidney level of triglycerides was significantly low in HCD vitamins treated groups (RT + HCD, AA + HCD and RT + AA + HCD), except in HCD + RT male group, as compared to HCD groups of rats (Table 7).

**Table 7:** Effect of rutin (RT) and/or ascorbic acid (AA) on kidney Total Cholesterol and Triglycerides levels in high-cholesterol diet (HCD) fed rats following 6 weeks of supplementation

Treatment (in rat chow)	Total Cholesterol (mg/g wet tissue)		Triglycerides (mg/g wet tissue)	
	
	Male	Female	Male	Female
**Control**	13.58 ± 1.72	16.75 ± 1.39	6.14 ± 0.94	6.42 ± 0.82

**RT (0.2%)**	12.99 ± 1.36	16.64 ± 1.24	6.29 ± 1.02	6.35 ± 1.10

**AA (0.4%)**	12.36 ± 1.12	16.42 ± 1.44	6.11 ± 0.80	6.18 ± 0.70

**RT (0.1%) + AA (0.2%)**	12.33 ± 0.46	16.72 ± 1.10	5.83 ± 0.54	6.00 ± 0.77

**HCD (1% cholesterol + 0.5% cholic acid)**	24.86 ± 2.80 ^a^	32.66 ± 4.53 ^a^	10.11 ± 1.11 ^a^	13.46 ± 1.19 ^a^

**RT (0.2%) + HCD**	21.87 ± 2.02 ^abe^	27.69 ± 2.18 ^abe^	9.40 ± 1.04 ^ab^	10.95 ± 1.96 ^abe^

**AA (0.4%) + HCD**	19.43 ± 0.83 ^ace^	23.28 ± 2.51 ^ace^	7.90 ± 0.59 ^ace^	8.45 ± 0.69 ^ace^

**RT (0.1%) + AA (0.2%) + HCD**	16.99 ± 1.41 ^ade^	20.59 ± 2.89 ^ade^	6.19 ± 0.83 ^e^	7.38 ± 0.78 ^e^

Data were expressed as Mean ± S.D and analyzed using one-way ANOVA followed by Student Newman-Keuls method as post hoc test. Six rats were used in each group. ^a^All treated groups vs. Control; ^b^RT + HCD vs RT group; ^c^AA + HCD vs AA group; ^d^RT + AA + HCD vs RT + AA group; ^e^RT + HCD, AA + HCD and RT + AA + HCD vs HCD

#### Histopathological evaluations

In male groups, histopathological examination revealed; (1) normal cortical and medullary pattern with well-formed glomeruli in control group, (2) hypercellular glomeruli with mesangeal cells proliferation till occlusion of glomeruli and few shrink as well as a swelling in tubular epithelial cells in HCD group, (3) proliferated hypercellular glomeruli with mesangial cells proliferation along with some swollen epithelial cells and no interstitial inflammatory cells in RT + HCD group, (4) hypercellular glomeruli with mesangeal cell proliferation and occasional some shrinked glomeruli with no tubulointerstitial inflammatory cells in AA + HCD group, (5) a preserved cortex and medulla with preserved normal looking glomeruli surrounded by benign tubules with slight swollen epithelail cells of some tubules in RT + AA + HCD group (Table 8 and Additonal file 1: Table S1). In female groups, histopathological examination revealed; (1) normal looking glomeruli with glomerular capillaries with no tubulointerstitial cell infiltrate in control group, (2) considerable number of hypercellular glomeruli with mesangeal proliferation of more than 50% along with tubular dilatation containing hyaline casts and epithelial swelling as well as few foci of tubulointerstitial inflammatory cell in HCD group, (3) scattered few hypercellular glomeruli with mesangeal cell proliferation as well as few eosinophilic hyaline casts within some tubules in RT + HCD group, (4) scattered hypercellular glomeruli less than 25% of glomeruli as well as few scattered tubulointerstitial cell infiltrates and few swollen epithelial cells of tubular epithelium in AA + HCD group, (5) in RT + AA + HCD group scattered few hypercellular glomeruli with mesangeal cell proliferation along with scattered few tubules containing eosinophilic hyaline casts with swollen epithelial cell lining (Table 8 and Additonal file 1: Table S1).

**Table 8:** Histopathological scoring of glomerular damage, acute tubular necrosis, tubulointerstitial nephritis as well as degree of nephrotoxicity after rutin (RT) and/or ascorbic acid (AA) treatments in high-cholesterol diet (HCD) fed rats following 6 weeks of supplementation

Treatment (in rat chow)	Glomerular damage		Acute tubular necrosis		Tubulointerstitial nephritis		Total score		Nephrotoxicity degree	
	
	Male	Female	Male	Female	Male	Female	Male	Female	Male	Female
**Control**	1	0	0	1	0	1	1	1	No	No

**HCD (1% cholesterol + 0.5% cholic acid)**	2	2	3	2	1	2	6	6	Moderate	Moderate

**RT (0.2%) + HCD**	1	1	1	1	0	0	2	2	Mild	Mild

**AA (0.4%) + HCD**	2	1	1	0	0	1	3	2	Mild	Mild

**RT (0.1%) + AA (0.2%) + HCD**	0	1	0	1	0	0	0	2	No	Mild

## Discussion

The effects of dietary rutin and/or ascorbic acid on high cholesterol diet (HCD) induced-nephrotoxicity in male and female Wistar albino rats were investigated. HCD administration for six consecutive weeks induced renal injury and nephrotoxicity, which was more in female than in male animals. Moreover, rutin and ascorbic acid were found to protect from HCD induced-nephrotoxicity. In addition, these nephro-protective effects were improved in our study by administration of both vitamins in combination to the HCD fed rats which was supported by results from histopathological investigation.

HCD is well known to cause nephrotoxicity and renal injury in different animal models. In the present study, signs of increased renal oxidative damage induced by HCD supplementation for six weeks were studied. Mean kidney weights were not significantly changed after HCD feeding and these results are in agreement with earlier reports [29,30]. This may be because of HCD could not alter the body weights significantly. Plasma creatinine levels also remained same in HCD group as compared to control animals. Similar results are reported by Kasiske et al. (1990) that, HCD supplementations to rats did not change the creatinine levels although kidney injury was reported [29]. Moreover, HCD feeding for eight weeks could not significantly altered the plasma creatinine levels in rats [31]. In our study, HCD administration to male as well as female rats resulted in moderate degree of nephrotoxicity as confirmed by the histopathological investigation. Treatment with HCD for six weeks significantly altered kidney levels of total cholesterol and triglycerides. Moreover, kidney level of MDA, a specific lipid peroxidation marker, was elevated while GSH level was decreased after six weeks of HCD diet administration to rats. Previous data showed HCD administration to cause hyperlipidemia and to be associated with oxidative stress and nitric oxide inactivation by ROS, which diminishes nitric oxide (NO) bioavailability leading to renal dysfunction [32]. Furthermore, HCD elevated brain, kidney and erythrocytes levels of lipid peroxidation products while decreased GSH content [33]. It was reported that HCD induces modification in lipid composition of cell membranes and the extracellular matrix to be more prone to free radical generation [34]. Therefore, we suggest that HCD induced-nephrotoxicity reported in our study were due to increased rate of oxidative stress and lipid peroxidation in the kidneys, which are kwon to potentiate generation of reactive oxygen species (ROS) and renal injury.

The present study also showed that HCD can induce more significant renal impairment and toxicity in female than male animals. This suggests that male antioxidant defense response to HCD-induced neohrotoxicity was higher than female response. It is well known that sexual steroid hormones can control many physiological and pathophysiological processes [35]. Sexual hormones have a selective influence on gene transcription and RNA production, which explain their regulation of the biosynthesis of specific proteins [36]. Therefore, these hormones can regulate endogenous pattern of antioxidant defense enzyme expression. Several studies have demonstrated the sexual dimorphism in the activity of antioxidant defense enzymes in different rat tissues [37-41]. One study conducted by Capel and Smallwood [42] did not find a noteworthy difference in glutathione peroxidase (GSH-Px), one of the antioxidant endogenous enzyme, activity in the brain between males and females, while in the liver the activity of this enzyme was significantly higher in females than in males. Similar results on the liver GSH-Px activity were noticed by in Prohaska and Sunde study [37]. Interestingly, Finley and Kincaid [43] reported that plasma and kidney cytosol selenium content, an endogenous antioxidant, and GSH-Px activity were increased in male compared to the same tissues in female animals, although both were increased in female liver cytosol than male. Experimental data confirmed that males exhibited significantly higher levels of endogenose antioxidant defense parameters in kidney such as Mn, Cu, Zn, SOD, glutathione peroxidase, and catalase than females [44]. Therefore, higher basal levels of ROS are measured by lucigenin chemiluminescence in the renal courtex of females [44]. Furthermore, total renal superoxide dismutase and catalase activities were found to be higher in male spontaneously hypertensive rats than females [44]. These findings are in agreement with our study results as male rats showed more renal antioxidant defense response to HCD-induced increase in MDA and decrease in RNA and total protein than did female rats.

One possible explanation of the degree of renal oxidative damage observed in female rats is that females are able to oxidize poly unsaturated fatty acids more than males. High fat diet was reported to reduce testosterone and to increase estrogen serum levels, which was associated with increased intra-abdominal fats in male than in female [32]. That is why exogenous treatment of ovariectomized rats with estrogen was shown to limit the ovariectomy-induced increase of adiposity [45]. In one study, male, but not female, rats showed a significant negative correlation with adipose poly unsaturated fatty acids content, which suggests that poly unsaturated fatty acids metabolism and oxidation is higher in female than in male [32]. This high rate of lipid metabolism and oxidation could influence more lipid peroxidation and generation ROS. Another possible mechanism is that estrogen itself is able to induced oxidative stress [46]. It was reported that, interaction between estrogen and its receptor (ER) involves an oxidative stress-mediated pathway [47]. Some studies indicate that estrogen exposition damages DNA under acute conditions in cell culture, which was more significant in ER positive cells [47,48]. Moreover, Wellejus and Loft 2002, suggested that the mechanism of estrogen induced oxidative stress may involve second messenger systems at which estrogen can bound to its cellular receptor and then transported to estrogen-sensitive genes in the nucleus, where redox cycling may take place [49].

In the present study, HCD induced renal impairment was significantly attenuated by administration of rutin and ascorbic acid. Our results revealed that the combination of both vitamins could have an additive, or sometimes synergistic, antioxidant effects. These speculations are supported by the histopathological evaluation that showed rutin and ascorbic acid combination to prevent HCD-induced nephrotoxicity especially in male animals. Flavonoids such as rutin are now widely accepted as physiologic antioxidants which are known to have effective free radical scavenging activity [50-52]. Previous data reported rutin, or vitamin P, to decrease the permeability of capillaries, scavenge free radicals, lower hepatic and blood cholesterol levels, and have antiplatelet activity [15,53,54]. Vitamin C (ascorbic acid) is a promising and effective agent in treating and preventing of allergic rhinitis [55], diabetes [56], heart disease [57] and cancer [58]. Measurements of kidney level of nucleic acids revealed that rutin and ascorbic acid combination can prevent renal cytotoxic damage induced by HCD supplementation to the rats. However, kidney level of total cholesterol results showed that both vitamins had a reno-protective effect that was similar when they were supplemented alone or in combination. Furthermore, HCD induced elevation in kidneys triglyceride was significantly attenuated by vitamins combination with an extent that was much better than administration of each vitamin alone. ROS are well known to induce cyto-toxicity and their action on unsaturated fatty acids has been implicated in the pathogenesis of various diseases [15]. Several studies reported both rutin and ascorbic acid to have a potent ability to damage free radicals produced through biological processes in many extracellular and intracellular reactions [15,59]. Therefore, we suggest that the combined renal cyto-protective effects of both rutin and ascorbic acid are due to their free radical scavenging capability which seems to be increased by both vitamins combination.

In addition, both vitamins combination significantly decreased MDA and elevate GSH kidney levels as compared to their altered levels in HCD group suggesting that the antioxidant properties of rutin may be attributed to its protective effects on lipid peroxidation. Rutin can augment cellular antioxidant defense mechanisms leading to the protection against oxidative tissue injury [15]. Rutin metabolism, inside the intestines by microflora, results in formation of its aglycone quercetin, which is responsible for rutin's in vivo antioxidant activity [60]. Previous data showed that rutin possess membrane lipid peroxidation inhibitory effects [13]. Furthermore, rutin was reported to increase the antioxidant capacity in the kidney of normal rats as well as in a liposomal model [61,62]. Rutin can prevent lipid peroxidation by chelating metal ions such as ferrous cations [60]. These ions are involved in the so called Fenton reaction, which generates ROS leading to lipid peroxidation. Interestingly, when red wine, which is known to contain rutin as one of its main components, was supplemented with HCD for 4 weeks, total cholesterol and lipid peroxidation products in the brain, kidney and erythrocytes were significantly decreased compared with the high-cholesterol diet alone, while GSH content and antioxidative enzymes activities were enhanced [33]. In parallel, vitamin C functions as an electron donor to protect the body from radicals and pollutants [63,64]. A study conducted by Grajeda-Cota et al. [65] reported that the extracellular ascorbic acid can scavenge ROS derived by the oxidation-reduction cycle which protected from tissue damages. Vitamin C is reported to improve diabetic nephro- and retino-pathy through its ability to induce remission of lipid peroxidation and lipid metabolism abnormalities in adult rats [59]. Indeed, the protection against of oxidative modification of proteins and the alteration in endogenous antioxidants activities such as glutathione might be trough the chelating property of vitamin C to react with free radicals or with highly reactive by products of lipid peroxidation [66].

## Conclusion

Taken together, we suggest that HCD can induce nephrotoxicity with higher extent in female than in male animals, suggesting a more antioxidant defense mechanism in kidneys of male than female animals. Moreover, we concluded that combining rutin and ascorbic acid can augmented their ability to attenuate ROS-induced lipid peroxidation which prevented HCD induce renal injury in Wistar albion rats.

## Competing interests

The authors declare that they have no competing interests.

## Authors' contributions

SS, HM and OA performed experimental work including animal treatments, biochemical analysis, statistical analysis, interpretation and discussion of the results related to their part of the work. SS and AM conceived, design and planning of the study, wrote the paper, drafting and revision of the manuscript. All authors read and approved the final manuscript.

## Supplementary Material

Additional file 1 **Table S1**. Effect of rutin (RT) and/or ascorbic acid (AA) on kidney histopathological evaluation in high-cholesterol diet (HCD) fed rats following 6 weeks of supplementation. Click here for file
