# Supplementary material for: Gender difference following high cholesterol diet induced renal injury and the protective role of rutin and ascorbic acid combination in Wistar albino rats
Source: Lipids Health Dis. 2012 Mar 16;11:41. doi: 10.1186/1476-511X-11-41 (PMC3352257; doi:10.1186/1476-511X-11-41)
Supplement: Additional file 1 — Table S1. Effect of rutin (RT) and/or ascorbic acid (AA) on kidney histopathological evaluation in high-cholesterol diet (HCD) fed rats following 6 weeks of supplementation. [file 1476-511X-11-41-S1.DOCX]

**Additional file 1:**

**Table S1** Effect of rutin (RT) and/or ascorbic acid (AA) on kidney histopathological evaluation in high-cholesterol diet (HCD) fed rats following 6 weeks of supplementation.

| **Treatment (in rat chow)** | **Male** | **Female** |
| --- | --- | --- |
| **Control** | 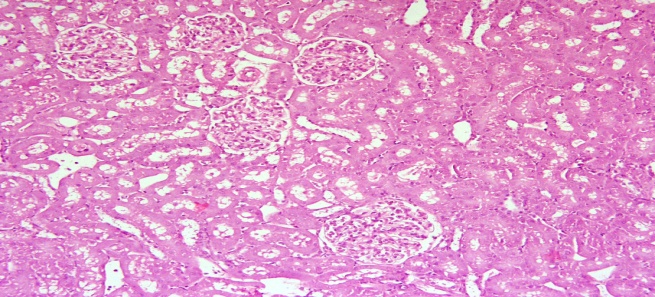 | 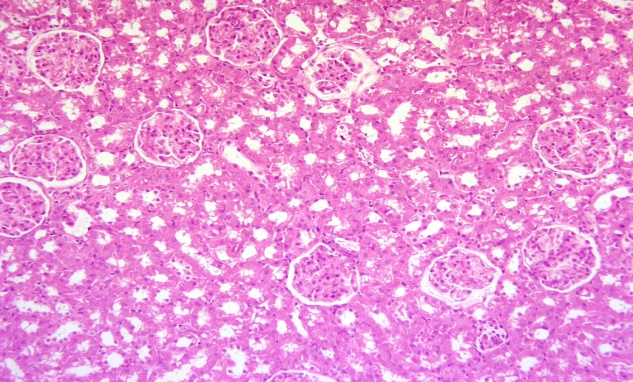[ |
| **HCD (1% cholesterol + 0.5% cholic acid)** | 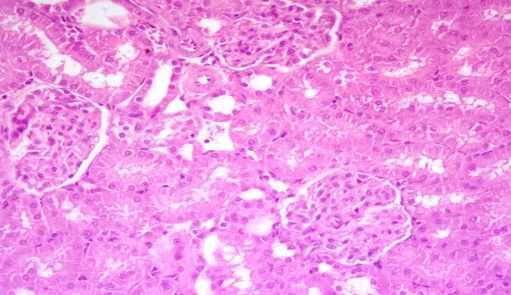 | 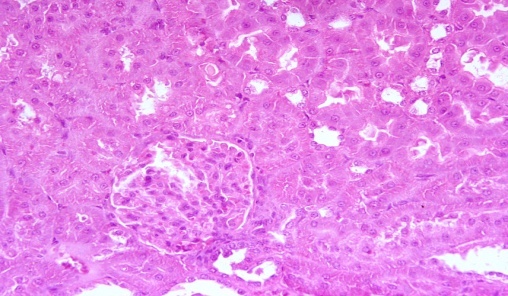 |
| **RT (0.2%) + HCD** | 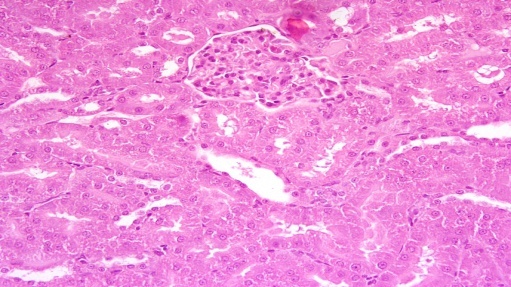 | 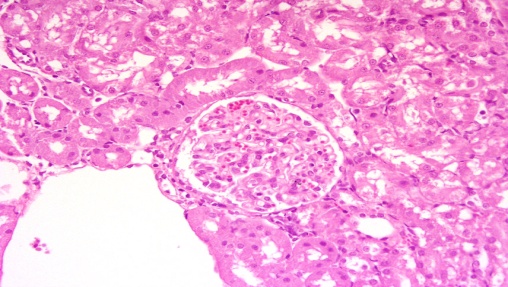 |
| **AA (0.4%) + HCD** | 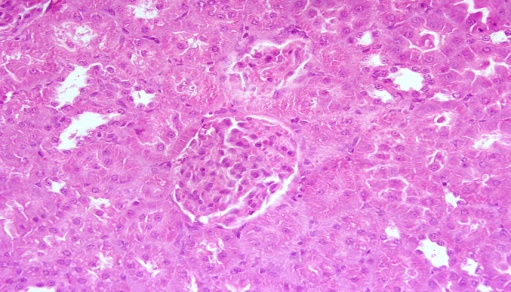 | 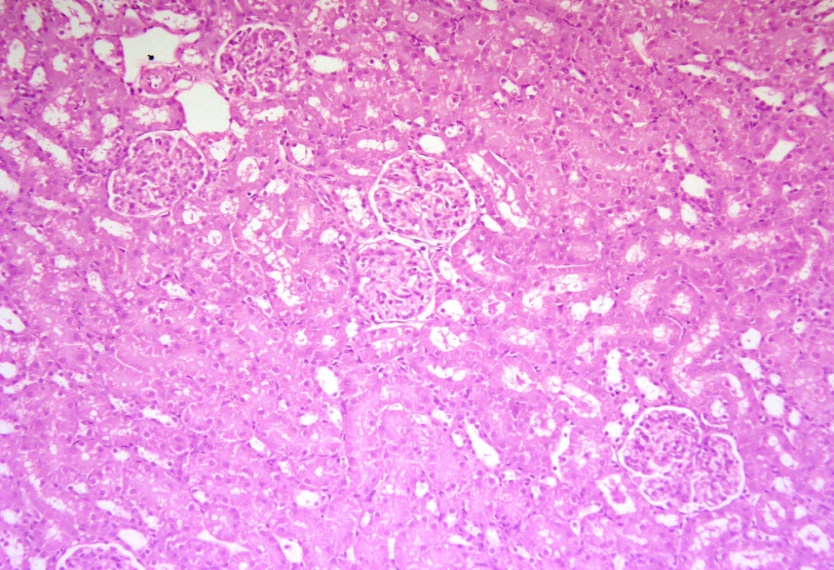 |
| **RT (0.1%) + AA (0.2%) + HCD** | 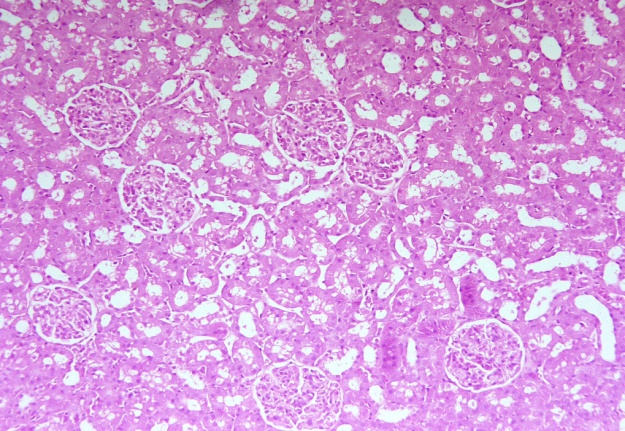 | 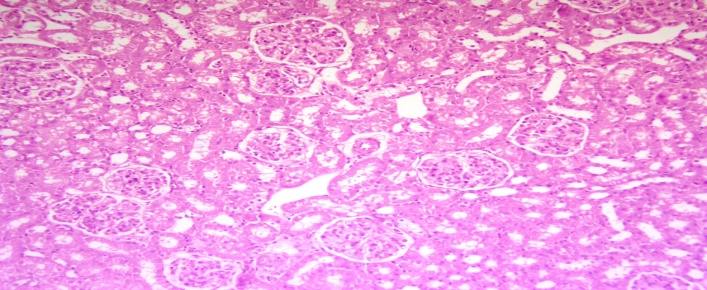 |

Histopathological sections revealed; no nephrotoxicity in male and female control groups, moderate nephrotoxicity in HCD male and female group, mild nephrotoxicity in RT+HCD and AA+HCD male and female groups as well as in RT+AA+HCD female group, no nephrotoxicity in RT+AA+HCD male group.
